# Persistent homology index as a robust quantitative measure of immunohistochemical scoring

**DOI:** 10.1038/s41598-017-14392-y

**Published:** 2017-10-25

**Authors:** Akihiro Takiyama, Takashi Teramoto, Hiroaki Suzuki, Katsushige Yamashiro, Shinya Tanaka

**Affiliations:** 1 Department of Cancer Pathology, Hokkaido University Graduate School of Medicine, Kita 15, Nishi 7, Kita-ku, Sapporo, Hokkaido 060-8638 Japan; 2 Department of Mathematics, Asahikawa Medical University, 2-1-1-1, Midorigaoka-higashi, Asahikawa, Hokkaido, 078-8510 Japan; 3 Division of Pathology, Hokkaido Cancer Center, 3-54, Kikusui 4-Jyou 2-Choume, Shiroisi-ku, Sapporo, Hokkaido, 003-0804 Japan

## Abstract

Immunohistochemical data (IHC) plays an important role in clinical practice, and is typically gathered in a semi-quantitative fashion that relies on some degree of visual scoring. However, visual scoring by a pathologist is inherently subjective and manifests both intra-observer and inter-observer variability. In this study, we introduce a novel computer-aided quantification methodology for immunohistochemical scoring that uses the algebraic concept of persistent homology. Using 8 bit grayscale image data derived from 90 specimens of invasive ductal carcinoma of the breast, stained for the replicative marker Ki-67, we computed homology classes. These were then compared to nuclear grades and the Ki-67 labeling indices obtained by visual scoring. Three metrics for IHC staining were newly defined: Persistent Homology Index (PHI), center coordinates of positive and negative groups, and the sum of squares within groups (WSS). This study demonstrates that PHI, a novel index for immunohistochemical labeling using persistent homology, can produce highly similar data to that generated by a pathologist using visual evaluation. The potential benefits associated with our novel technology include both improved quantification and reproducibility. Since our method reflects cellularity and nuclear atypia, it carries a greater quantity of biologic data compared to conventional evaluation using Ki-67.

## Introduction

Breast cancer is the most commonly diagnosed cancer in Japanese women^1^, for which invasive ductal carcinoma is the most frequent type of invasive carcinoma. Its histological or nuclear grading provides useful data for predicting prognoses^2,3^. Ki-67, a nuclear protein and cellular marker of proliferation, is used as a predictive and prognostic marker in many cancers. Multiple studies have shown that a higher proliferation rate (i.e. elevated staining of Ki-67) is associated with a poor prognosis^4–6^. Further, the St. Gallen Consensus (2009) states that the Ki-67 labeling index is a useful parameter with which to select patients with hormone receptor-positive breast cancers who may benefit from chemotherapy as well as endocrine therapy. Breast cancers are classified as low, intermediate, or highly proliferative according to their Ki-67 labeling index, with these categories linked to labeling indices of under 15%, 16–30%, or over 30%, respectively^7^. On the other hand, gene expression profiling enabled us to classify breast tumors under five intrinsic subtypes, i.e., luminal A, luminal B, HER2 over-expression, basal and normal-like tumors^8,9^. This classification is approximated by information of ER, PgR, HER2, and Ki-67^10,11^. However, since the evaluation of Ki-67 labeling is itself not standardized, and visual scoring is used in many facilities, index values vary considerably.

In this study, we propose a novel quantitative evaluation method for immunohistochemical labeling based on persistent homology. Persistent homology is a relatively new algebraic tool developed from a practical standpoint (details are provided in the section of Methods). In terms of shape recognition in image analysis, the classical tools for capturing the characteristics of shape from black and white images are sensitive to noise in data sets. Coping with this difficulty, persistent homology enables us to measure stable topological features in a meaningful manner. The presumption is that stable topological features (and by inference the most valuable) are revealed by their persistence. In this work, we investigated the correlation between the newly defined persistent homology-derived index, nuclear grade, and Ki-67 labeling index, as obtained by traditional visual scoring.

## Methods

### Patients

Ethical approval for this study was obtained from the institutional review board (IRB) of the Hokkaido Cancer Center and Asahikawa Medical University, which waived the need for informed consent. All experiments and methods were performed in accordance with relevant guidelines and regulations. We selected 90 cases of invasive ductal carcinoma of the breast that were surgically resected at Hokkaido Cancer Center, Sapporo, Japan, from January 2015 through September 2015. The median patient age was 60 (range 25–87). 44 patients were diagnosed with scirrhous carcinoma, 41 with papillotubular carcinoma, and 5 with solid-tubular carcinoma. These 90 patients comprised 56 cases of tumors assigned the Japanese nuclear grade of 1, 19 cases of grade 2, and 15 cases of grade 3.

The Japanese nuclear grade is defined as the sum of two parameters, namely nuclear atypia (l for low-degree atypia; 2 for intermediate-degree atypia; 3 for high-degree atypia), and mitotic count/10 high power fields (x40 objective lens). A score of l denotes 0–4 mitoses. Two indicates 5–9 mitoses, and 3, ≥10 mitoses. To derive a nuclear grade we then sum the scores (g) for the two parameters. If g equals 2 or 3, then the nuclear grade is designated 1. For a g score of 4, the nuclear grade is 2. Grade 3 is reserved for g scores of 5 or 6^3^. An initial assessment of Ki-67 staining was made at × 40 magnification, with the most intense fields selected for assessment using a Nikon ECLIPSE 80i type microscope (for which a × 40 field size corresponds to 0.0625mm^2^). Sections were scanned using a Scorpion IEEE-1394 camera (Point Grey Research^®^ Inc.) with images saved in TIFF format (1280 × 960 pixels). The Ki-67 labeling index was recorded from the original pathology report. If this number was given as a range (e.g. 30–40%), then we used the mean value (35% in the example given) for statistical analyses. Scanned images (magnification: × 40, total images: 90) were processed using Apple MacPro (3.0 GHz 8Core).

### Immunohistochemistry

Sections were cut at 3 to 4μm intervals and immuno-stained using the monoclonal antibody Ki-67 (clone 30-9, pre-diluted, Ventana-Roche, Arizona, USA). Immunohistochemical stains were subsequently performed using Bench Mark GX with the iView DAB Detection kit (Ventana-Roche, Arizona, USA).

### Persistent Homology

Persistent homology is a fairly recent mathematical concept (reviewed^12–17^). Let *M* be a grayscale image of size *X* × *Y* with gray intensities 2^*n*^ (in our case, *n* = 8 (8 bit grayscale)). *M* is considered to be an integer-valued function of two variables *f*(x, y) on the intervals [0, 2^*n*^ − 1]. Let *K*_*i*_ denote the sublevel set *f* ^−1^([0, *i*]) of the function *f*. For each *i*, *j* $(0\le i\le j\le {2}^{n}-1)$, we have a sequence *K*:

1 $${K}_{0}\subset {K}_{1}\subset {K}_{2}\subset \cdots \subset {K}_{i}\subset \cdots \subset {K}_{j}\subset \cdots \subset {K}_{{2}^{n}-1},$$

which is called a sublevel set filtration. For each ${K}_{i}$, we can calculate the *p*-th homology group ${H}_{p}({K}_{i})$ and *p*-th Betti number ${\beta }_{p}({K}_{i})$ ^18^. An element of ${H}_{p}({K}_{i})$ is called a homology class. Intuitively, ${\beta }_{0}$ counts the number of connected components, and ${\beta }_{1}$ counts the number of “holes” or “voids”. For ${K}_{i}\subset {K}_{j}$, the image of the induced homomorphism ${f}_{p}^{i,j}:{H}_{p}({K}_{i})\to {H}_{p}({K}_{j})$ is called a persistent homology group, and its rank ${\beta }_{p}^{i,j}=rank\{im({f}_{p}^{i,j})\}$ is called a persistent Betti number of *f*. We can track the birth (*i*) and death (*j*) of each homology class (feature) during the evolution of this sequence, which can be represented using the interval $[i,j]$, which is called the persistence interval.

Persistent Betti numbers are visualized by plotting $(i,j)\in {Z}^{2}$, i.e., [0,255]^2^ with multiplicity given by ${\mu }_{p}^{i,j}={\beta }_{p}^{i,j-1}-{\beta }_{p}^{i-1,j-1}-({\beta }_{p}^{i,j}-{\beta }_{p}^{i-1,j})$ at each point in two dimensions. This plot, according to the birth and death of two dimensional coordinates is called a *p*-th persistence diagram (PD_*p*_), in which all points lie above the diagonal, i.e, *i* < *j*, and the vertical distance to the diagonal indicates the persistence of *p*-dimensional holes. Those points close to the diagonal (i.e. short vertical bars): {$(i,j)$ in PD_*p*_ | |*j* − *i*| < δ} are classified as topological noise artifacts; long distances from the diagonal (i.e. persistent) represent more robust features. In this study, we assigned a value of 40 for δ. We computed persistence diagrams (PD_*p*_) from 8 bit grayscale image data using the open source software, Persistent Homology Algorithm Toolbox (PHAT, v1.4.1)^19^, and used MATLAB R2015b to draw persistence diagrams. To improve the visibility of the persistence diagrams, we divided them into 32^2^ grids and demonstrated the multiplicity in each grid by using color gradients.

### Simple Example of a Persistence Diagram

**Figure 1:**
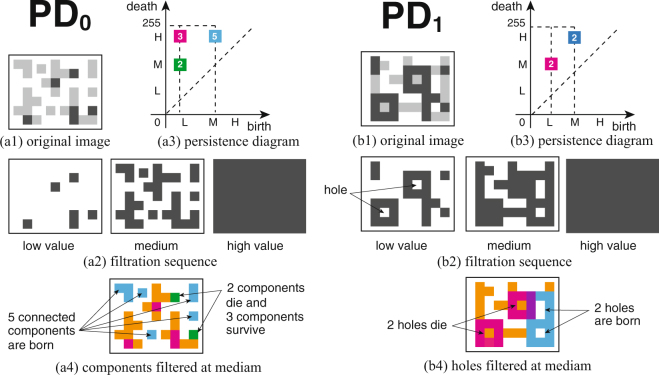
Conceptual schema of the filtration and persistence diagram for pixel image data. For simplicity we assume that there are only 3 grayscale levels, namely, black (low, L), gray (medium, M), and white (high, H), respectively. The figures show the case of zeroth and first persistence diagram (PD_0_ and PD_1_) for connected components and holes, respectively. We generate the filtration sequences (**a2** and **b2**) of the images by increasing the grayscale threshold from a low to high value, corresponding to from the original image of (**a1** and **b1**) to the background. The persistence diagrams of (**a3**) and (**b3**) are obtained by plotting the locations of the persistent Betti numbers ${\beta }_{p}^{i,j}\,\,$as a point $(i,j)\in {Z}^{2}$. The white numbers on each square indicate the multiplicities at each point (i, j). (**a4** and **b4**) Schematic image of the colored blocks which generate the associated persistent Betti numbers ${\beta }_{p}^{i,j}$ on the persistence diagrams PD_p_ at the medium level. The details are given in Methods.

Figure 1 shows schematics that demonstrate a *p*-th persistence diagram PD_*p*_, with *p* = 0 for connected components, and *p* = 1 for holes, respectively. For simplicity we assume that there are only 3 levels of grayscale, namely, black (low, L), gray (medium, M), and white (high, H), corresponding to positive cells, negative cells, and background, respectively (Fig. 1(a1) and (b1)). We generated the filtration sequence for the pixel images by increasing the grayscale threshold from low to high values (Fig. 1(a2) and (b2)). In Fig. 1(a3) and (b3), the persistent Betti numbers ${\beta }_{p}^{i,j}$ are plotted as the colored squares in two-dimensional space with respect to the (birth, death)-coordinates. The white number on each square shows the multiplicities ${\mu }_{p}^{i,j}$ at a point (i, j) on the persistence diagram. Fig. 1(a4) shows the schematic image at the medium filtration level, in which each color of blocks are associated with the color of squares in (a3). Both of green- and magenta-colored blocks appear in the lower filtration level. Each 2 green-colored blocks are connected to the magenta-colored blocks through the orange-colored blocks, and then, they merge into the connected domains. We suppose that the green-colored blocks are adsorbed into the connected domain with the corresponding magenta-colored blocks. That is, the 2 green-colored blocks die at the medium filtration level and the 3 magenta-colored blocks survive to the higher level. Hence we put a green square at the point (L, M) with a multiplicity number 2, which means that the persistence intervals for the lower cells are [L, M]. Moreover we put a magenta square at the point (L, H), i.e., the persistence intervals for the upper cells are [L, H] with multiplicity number 3. On the other hand, the cyan square at the point (M, H) in (a3) indicates the 5 cyan-colored blocks which newly appear at the medium level in Fig. 1(a4). The persistence interval is [M, H] and the multiplicity number is 5. For the case of PD_0_ (Fig. 1(a3)), the zeroth persistent Betti numbers associated with positive and negative cells appear in the lower and higher birth value regions in the diagram, indicated by the magenta- and cyan-colored squares, respectively. For the case of PD_1_ (Fig. 1(b3)), the first persistent Betti numbers indicate the robustness of each hole. The white numbers depicted on colored squares indicate the multiplicities ${\mu }_{1}^{i,j}$ at each point (i, j) on the (birth, death)-coordinates. Fig. 1(b4) shows the schematic image colored at the medium filtration level. The magenta-colored blocks surround 2 holes at the lower level, and then these 2 magenta-colored holes disappear at the medium level by being filled with the orange-colored blocks, i.e., the persistence interval of the 2 magenta-colored holes is [L, M]. Therefore, we put a magenta square at the point (L, M) with the multiplicity number 2 in (b3). Moreover, the 2 cyan-colored holes newly appear and survive to the higher level. We put a cyan square at the point (M, H) corresponding to the persistence interval [M, H] with the multiplicity number 2. The purple-colored blocks in (b4) indicate the overlapped part of the magenta-colored and cyan-colored blocks.

### Persistent Homology Index

**Figure 2:**
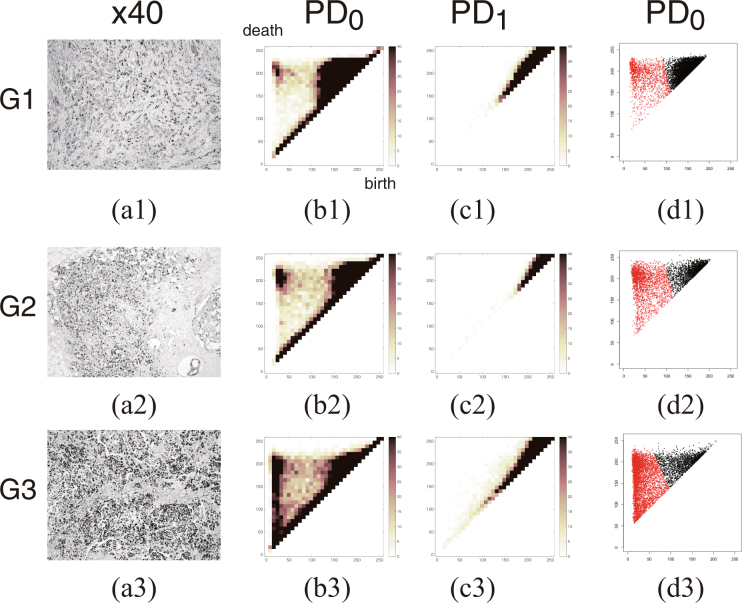
Representative persistence diagrams of Ki-67 staining for the three different nuclear grades of tumor. Representative 8-bit grayscale images of Ki-67 (×40 magnification) for Grade 1, Grade 2, and Grade 3 tumors (**a1**–**a3**). The zeroth and first persistence diagrams PD_0_ and PD_1_, represented by (**b1**–**b3**) and (**c1**–**c3**), denote the space [0,255]^2^ divided into 32^2^ grids, with the multiplicity of persistent Betti numbers at each grid shown using the color gradient [0, 40]. The persistent Betti numbers for positive and negative cell groups are plotted in the two dimensional space of [0,255]^2^ using red and black colored points, respectively in (**d1**–**d3**). The Betti numbers corresponding to Ki-67 positive and negative cells appear in the upper-left and upper-right regions of the persistence diagram (PD_0_). With advancing nuclear grade, the number of positive cells increases.

The nuclei of the Ki-67 positive cells have smaller birth parameters and larger death parameters in the persistence diagram PD_0_; negative cells exhibit larger birth and death parameters. Therefore the homology classes of positive cells are distributed at the upper left hand region of the persistence diagram, with those of negative cells at the upper right hand region. The homology classes on the persistence diagrams are classified into two clusters using the k-means clustering algorithm^20^. In Fig. 2(d1–d3), we plot the persistent Betti number ${\beta }_{0}^{i,j}$ in the original 256^2^ grids using red and black colored points. The persistent Betti number ${\beta }_{0}^{i,j}$ associated with positive (resp. red) and (resp. black) negative cells appear in the upper-left and upper-right regions of PD_0_. Let *C*_*1*_ and *C*_2_ be cluster centers of two clusters, such that the birth-parameter of *C*_1_ is smaller than that of *C*_2_ (noting that the positively stained cells have smaller birth parameters). The homology classes that induce *C*_*1*_ are called positive, and those that induce *C*_*2*_ are called negative. Center_*x*1_ and Center_*y*1_ denote the coordinates of *C*_*1*_, and Center_*x*2_ and Center_*y*2_ denote the coordinates of *C*_*2*_. The persistent homology index (PHI) is defined by the quotient of the total number of positive homology classes divided by the total number of positive and negative homology classes.

2 $${\rm{P}}{\rm{H}}{\rm{I}}\,:=\frac{{\rm{t}}{\rm{o}}{\rm{t}}{\rm{a}}{\rm{l}}\,{\rm{n}}{\rm{u}}{\rm{m}}{\rm{b}}{\rm{e}}{\rm{r}}\,{\rm{o}}{\rm{f}}\,{\rm{p}}{\rm{o}}{\rm{s}}{\rm{i}}{\rm{t}}{\rm{i}}{\rm{v}}{\rm{e}}\,{\rm{h}}{\rm{o}}{\rm{m}}{\rm{o}}{\rm{l}}{\rm{o}}{\rm{g}}{\rm{y}}\,\,{\rm{c}}{\rm{l}}{\rm{a}}{\rm{s}}{\rm{s}}{\rm{e}}{\rm{s}}}{{\rm{t}}{\rm{o}}{\rm{t}}{\rm{a}}{\rm{l}}\,{\rm{n}}{\rm{u}}{\rm{m}}{\rm{b}}{\rm{e}}{\rm{r}}\,{\rm{o}}{\rm{f}}\,{\rm{p}}{\rm{o}}{\rm{s}}{\rm{i}}{\rm{t}}{\rm{i}}{\rm{v}}{\rm{e}}\,{\rm{a}}{\rm{n}}{\rm{d}}\,{\rm{n}}{\rm{e}}{\rm{g}}{\rm{a}}{\rm{t}}{\rm{i}}{\rm{v}}{\rm{e}}\,({\rm{a}}{\rm{l}}{\rm{l}})\,{\rm{h}}{\rm{o}}{\rm{m}}{\rm{o}}{\rm{l}}{\rm{o}}{\rm{g}}{\rm{y}}\,{\rm{c}}{\rm{l}}{\rm{a}}{\rm{s}}{\rm{s}}{\rm{e}}{\rm{s}}},$$

Although this index resembles the Ki-67 labeling index it is actually extracted from a more sophisticated dataset insofar as the results are informed by cellularity and nuclear atypia, as well as by Ki-67 labeling.

The sum of squares between groups (BSS) represents the sum of the squares of the distances between the overall center, and the centers of all groups. The sum of squares within groups (WSS) is given by the sum of squares between the overall center within each group, and all points within the group. WSS1 denotes the WSS of a positive group, with WSS2 denoting a WSS of negative groups. The ratio of the sum of squares (RSS) is defined by ${\rm{RSS}}\,:=\frac{{\rm{BSS}}}{{\rm{BSS}}+{\rm{WSS}}1+{\rm{WSS}}2}$. The larger the RSS becomes, the clearer the division between groups.

### Statistical Analyses

Persistent homology indices and other data were compared with nuclear grade and the standardized Ki-67 labeling index (mean percentage) as extrapolated from pathology reports. Spearman correlation and Kruskal-Wallis tests were performed using statistical software R version 3.2.0^21^.

### Data Availability

The datasets generated and analyzed during the current study are available from the corresponding author on reasonable request.

## Results

### Persistence Diagrams

Figure 2 shows representative persistence diagrams of Ki-67 staining for the three different nuclear grades (Grade 1–Grade 3). Fig. 2(b1–b3 and c1–c3) show the zeroth and first persistence diagrams (PD_0_ and PD_1_) in which the space of [0,255]^2^ is divided into 32^2^ grids, with the multiplicity of persistent Betti numbers at each grid indicated using the color gradient [0, 40]. The dark colored region indicates a high multiplicity of persistent Betti numbers. In Fig. 2(d1–d3), persistent Betti numbers of positive and negative cell groups are plotted using red and black colored points, respectively. The zeroth persistence diagrams PD_0_ of (d1-d3) is shown in two-dimensional space of [0,255]^2^. The Betti numbers corresponding to Ki-67 positive and negative cells appear in the upper left-hand side of the PD_0_ diagram, and upper right-hand side, respectively. With advancing nuclear grade, the number of positive cells increases.

### Persistent Homology Index and the other indices obtained from the persistence diagrams PD_0_ and PD_1_ versus visual scoring of Ki-67 labeling index (LI) and nuclear Grades

**Figure 3:**
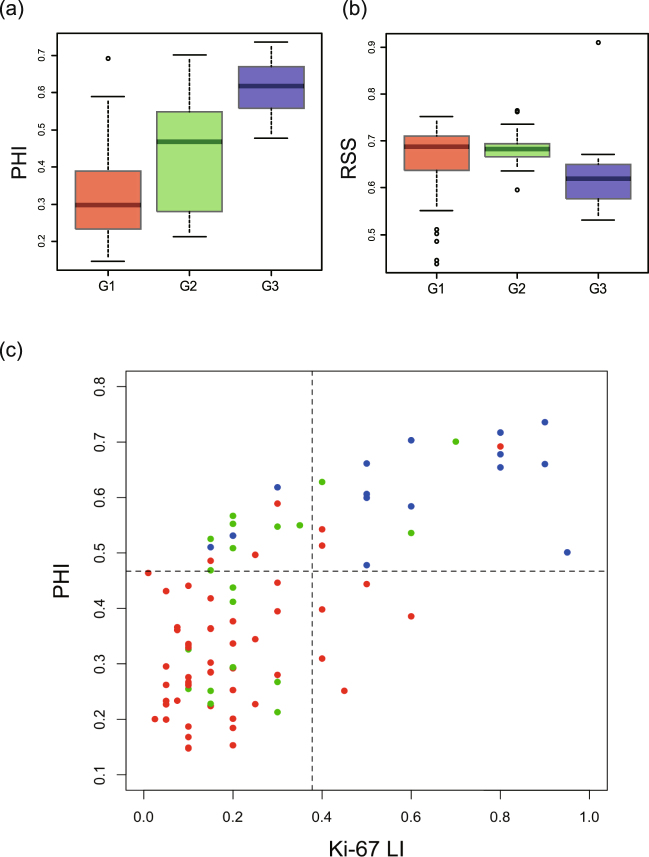
(**a**,**b**) Persistent Homology Index (PHI) and RSS versus visual scoring of nuclear grade and (**c**) Persistent Homology Index (PHI) versus visual scoring of Ki-67 labeling index (LI). Box plots of (**a**) PHI and (**b**) RSS using persistence diagrams PD_0_ (vertical axis) versus pathologist-derived (visual scoring) nuclear grade (G1, G2 and G3 shown in horizontal axis), p < 0.0001 (Kruskal-Wallis rank sum test) for PHI, and p = 0.0040 (Kruskal-Wallis rank sum test) for RSS. (**c**) A scatter diagram for PHI versus the pathologist-derived visual scoring of the Ki-67 labeling index (LI). These data revealed a high correlation between PHI and visual scoring (Spearman correlation 0.71, p < 0.0001). The red, green, and blue colors correspond to the nuclear grade G1, G2, and G3, respectively. The vertical and horizontal lines indicate the threshold values for the binary classification with respect to Ki67-LI and PHI, respectively.

Figure 3(a) and (b) show a box plot of the persistent homology index (PHI) and RSS, using the persistence diagram (vertical axis), versus a pathologist-derived visual score of nuclear grade (horizontal axis). We found that PHI was positively associated with nuclear grade (p < 0.0001: Kruskal-Wallis rank sum test). Mean values ± 95% CIs are provided in Table 1.

**Table 1:** Examples of indices calculated from the zeroth persistence diagram PD_0_.

	Grade	Mean	95% CI
PHI	G1	0.324	0.292–0.356
	G2	0.435	0.362–0.508
	G3	0.616	0.570–0.662
RSS	G1	0.663	0.642–0.683
	G2	0.684	0.663–0.704
	G3	0.629	0.580–0.678
WSS1	G1	42.00	39.84–44.17
	G2	42.97	39.00–46.94
	G3	41.57	37.91–45.23
WSS2	G1	26.57	25.23–27.90
	G2	29.00	26.57–31.42
	G3	33.96	31.61–36.32
${{\rm{Center}}}_{x1}$	G1	52.13	47.83–56.42
	G2	43.76	39.15–48.37
	G3	34.49	31.56–37.42
${{\rm{Center}}}_{y1}$	G1	175.1	169.0–181.3
	G2	170.7	155.3–186.0
	G3	137.4	121.1–153.6
${{\rm{Center}}}_{x2}$	G1	147.3	142.6–152.0
	G2	146.3	137.0–155.6
	G3	129.6	110.7–148.5
${{\rm{Center}}}_{y2}$	G1	206.6	203.8–209.5
	G2	205.9	199.8–212.0
	G3	193.9	182.4–205.4

Figure 3(c) shows a scatter diagram for Persistent Homology index (PHI) versus the pathologist-derived visual scoring of the Ki-67 labeling index (LI). These data revealed a high correlation between PHI and visual scoring of Ki-67 LI (Spearman correlation 0.71, p < 0.0001). In the diagram, the red-colored points of G1 (resp. blue colored-points of G3) appear in the left-bottom (resp. right-upper) regions, respectively. We estimate the accuracy rate for binary classification with respect to the Ki-67 LI and PHI, respectively. We categorize total data points for 90 patients into two types of grades, i.e., 56 points of G1 (type A) and 34 points of G2 and G3 (type B). This binary categorization is often taken account in determining the chemotherapy treatments with the classification to luminal A and B types. Applying the binary logistic regression with respect to Ki-67 LI, we obtain the threshold value for the binary classification as 0.378 (indicated by the vertical line in the figure). Testing the threshold value for 56 data points of type A (resp. 34 points of type B), the 48 points (resp. 15 points) are correctly classified into type A (resp. type B). Hence the accuracy rate for the Ki-67 LI is 0.700 ( = 63/90). In the same manner, we obtain the threshold value with respect to PHI as 0.467 (indicated by the horizontal line in the figure). For 56 data points of type A (resp. 34 points of type B), the 50 points (resp. 25 points) are correctly classified into type A (resp. type B). The accuracy rate for the PHI is 0.833 ( = 75/90). It is remarkable that the sensitivity for the binary classification is clearly improved from 15/34 to 25/34 by replacing Ki-67 LI into PHI.

**Figure 4:**
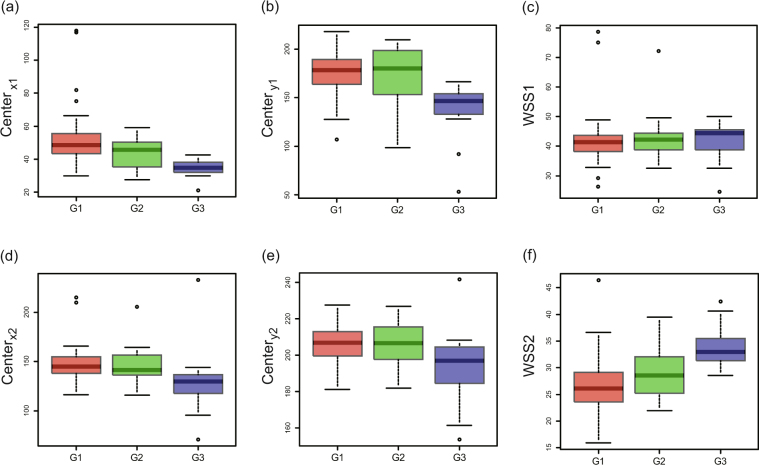
Indices obtained from the zeroth persistence diagram PD_0_ versus visual scoring of nuclear grade for positive cells (**a**–**c**) and negative cells (**d**–**f**). Box plots of (**a**) ${{\rm{Center}}}_{x1}$, (**b**) ${{\rm{Center}}}_{y1}$, and (**c**) WSS1 from the zeroth persistence diagrams PD_0_ for positive cells versus pathologist-derived (visual scoring) nuclear grade (G1, G2 and G3 in horizontal axis), p < 0.0001(Kruskal-Wallis rank sum test) for ${{\rm{Center}}}_{x1}$ and ${{\rm{Center}}}_{y1}$, and p = 0.7003(Kruskal-Wallis rank sum test) for WSS1. Box plots of (**d**) ${{\rm{Center}}}_{x2}$, (**e**) ${{\rm{Center}}}_{y2}$, and (**f**) WSS2 from the zeroth persistence diagrams PD_0_ for negative cells versus pathologist-derived (visual scoring) nuclear grade (G1, G2 and G3 in horizontal axis), p = 0.00064 (Kruskal-Wallis rank sum test) for ${{\rm{Center}}}_{x2}$, p = 0.01442(Kruskal-Wallis rank sum test) for ${{\rm{Center}}}_{y2}$, and p < 0.0001(Kruskal-Wallis rank sum test) for WSS2.

Figure 4(a–c) illustrate the characteristics of a group of positive cells, and shows the box plot of ${{\rm{Center}}}_{x1}$, ${{\rm{Center}}}_{y1}$, and WSS1 from the persistence diagram (vertical axis) versus a visual score of nuclear grade (again, on the horizontal axis). ${{\rm{Center}}}_{x1}$ and ${{\rm{Center}}}_{y1}$ were found to be negatively associated with nuclear grade (p < 0.0001: Kruskal-Wallis rank sum test). There is no significant difference in WSS1 (p = 0.7003: Kruskal-Wallis rank sum test). Mean values ± 95% CIs are shown in Table 1.

Figure 4(d–f) illustrate a similar treatment for the group of negative cells, with a box plot of ${{\rm{Center}}}_{x2}$, ${{\rm{Center}}}_{y2}$, and WSS2 from the persistence diagram (vertical axis), versus the pathologist-derived visual scores of nuclear grade (horizontal axis). ${{\rm{Center}}}_{x2}$ and ${{\rm{Center}}}_{y2}$ were negatively associated with nuclear grade (p = 0.00064: Kruskal-Wallis rank sum test; p = 0.01442: Kruskal-Wallis rank sum test, respectively), with WSS2 positively associated with nuclear grade (p < 0.0001:Kruskal-Wallis rank sum test). Mean values ± 95% CIs are shown in Table 1.

**Figure 5:**
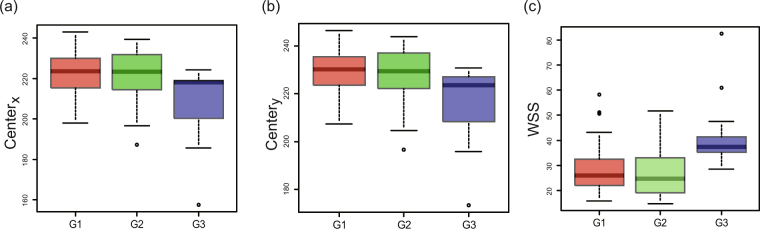
Indices of ${{\rm{Center}}}_{x}$, ${{\rm{Center}}}_{y}$, and WSS versus visual scoring of nuclear grade from the first persistence diagram PD_1_. Box plots of ${{\rm{Center}}}_{x}$, ${{\rm{Center}}}_{y}$, and WSS using persistence diagrams PD_1_ (vertical axis) versus pathologist-derived (visual scoring) nuclear grade (G1, G2 and G3 in horizontal axis). ${{\rm{Center}}}_{x}$ and ${{\rm{Center}}}_{y}$ are both negatively associated with nuclear grade (p = 0.00019:Kruskal-Wallis rank sum test; p = 0.00413:Kruskal-Wallis rank sum test, respectively), whereas WSS is positively associated with nuclear grade (p = 0.00023:Kruskal-Wallis rank sum test). Examples of indices calculated from the zeroth persistence diagram PD_0_: Here we evaluate the mean values and the associated confidence intervals (mean ± 95% CI) for PHI and RSS corresponding to Fig. 3, ${{\rm{Center}}}_{x1}$ and ${{\rm{Center}}}_{y1}$, and the square-root of WSS1 corresponding to Fig. 4(a–c) for positive cells, and ${{\rm{Center}}}_{x2}$ and ${{\rm{Center}}}_{y2}$, and the square-root of WSS2 corresponding to Fig. 4(d–f) for negative cells. Examples of indices calculated from the first persistence diagram PD_1_: Here we show the values of ${{\rm{Center}}}_{x}$ and ${{\rm{Center}}}_{y}$, and square-root of WSS to evaluate their mean values and the associated confidence intervals (mean ± 95% CI), corresponding to Fig. 5.

Figure 5 and Table 2 illustrate the characteristics for PD_1_ using a box plot of ${{\rm{Center}}}_{x}$, ${{\rm{Center}}}_{y}$, and WSS using the persistence diagram PD_1_ (vertical axis), versus the pathologist-derived visual scoring of nuclear grade (horizontal axis). ${{\rm{Center}}}_{x}$ and ${{\rm{Center}}}_{y}$ are both negatively associated with nuclear grade (p = 0.00019:Kruskal-Wallis rank sum test; p = 0.00413:Kruskal-Wallis rank sum test, respectively), whereas WSS is positively associated with nuclear grade (p = 0.00023:Kruskal-Wallis rank sum test). Mean values ± 95% CIs are shown in Table 2.

**Table 2:** Example of indices calculated from the first persistence diagram PD_1_.

	Grade	Mean	95% CI
${{\rm{Center}}}_{x}$	G1	222.8	220.2–225.4
	G2	221.3	214.7–227.8
	G3	207.4	197.5–217.4
${{\rm{Center}}}_{y}$	G1	229.2	226.9–231.5
	G2	227.6	221.7–233.5
	G3	216.0	207.3–224.7
WSS	G1	28.32	25.93–30.71
	G2	27.59	22.45–32.73
	G3	41.36	33.70- 49.02

## Discussion

Computational topology is a new discipline that includes methods for calculating topological invariants such as homology groups and Betti number from digital images. Various applications for this discipline may be found in areas of materials science. For example, Hiraoka *et al*. recently discovered hierarchical structures of amorphous solids using persistent homology^22,23^. The homology concept can be applied to the geometrical characterization of 2-dimensional fracture surfaces and 3-dimensional morphologies in polymer mixtures^24,25^. In medical sciences, Adcock *et al*. used persistent homology to classify liver lesions in CT images^26^. Nakane and Takiyama also applied homology methods for the identification of regions of interest in colonic pathological images^27^. Ferri *et al*. generated a feasibility study for a persistent homology based k-Nearest Neighbor search algorithm in melanoma detection^28^.

In this study, we have defined a persistent homology index (PHI). Comparisons of PHI versus the Ki-67 labeling index derived by a pathologist using visual scoring demonstrated a high Spearman correlation of 0.71 (p < 0.0001). Positive correlations were observed between PHI, WSS2, WSS, and nuclear grade, as assessed by visual scoring; negative correlations were observed for center coordinates. There were significant differences among the three groups of metric corresponding to each nuclear grade in PHI, center coordinates (except for ${{\rm{Center}}}_{y2}$), and WSS2 (p < 0.001) for the zeroth persistence diagram PD_0_, and center coordinates and WSS for the first persistence diagram PD_1_ (p < 0.01). The PHI, our novel index for immunohistochemical scoring using persistent homology, generated highly similar data that produced by visual scoring.

Positive correlations between WSS2, WSS and nuclear grade, and negative correlations between center coordinates and nuclear grade, indicate that persistence diagrams reflect not only labeling index, but also cellularity and nuclear atypia. The denser cellularity becomes, the earlier homology classes contact each other, such that the center of positive homology classes moves to a lower position. Nuclear atypia, such as differences in the size of nuclei and their irregular distribution, provoke this shift in the persistence diagram. These changes appear in ${{\rm{Center}}}_{x1}$ and ${{\rm{Center}}}_{y1}$ data. Since earlier contacts of homology classes bring about earlier births in the PD_1_ diagram, these changes also appear in ${{\rm{Center}}}_{x}$, ${{\rm{Center}}}_{y}$, and WSS calculated from PD_1._ Histological grading or nuclear grading of invasive ductal carcinoma is a useful prognostic factor^2,3,29,30^. Higher nuclear grades are linked to poorer prognoses, with many studies indicating a high correlation between Ki-67 labeling index and prognosis^4^. However, histological grading or nuclear grading by visual scoring is inherently subjective and manifests intra-observer and inter-observer variabilities^31^, with these exacerbated by the lack of a standardized Ki-67 labeling index^32^. Our new persistent homology index is objective and therefore provides more reliable data. Our method may provide a means to improve IHC data quality, with persistence diagrams carrying more information than the Ki-67 labeling index alone. Furthermore, our quantitative analyses can be applied to other immunohistochemical nuclear stains such as estrogen receptor (ER) and progesterone receptor staining (PgR). The quantification of immunohistochemical membrane staining, such as human epidermal growth factor receptor 2 (HER2), represents another possible application as this provokes as increase in 1-dimensional Betti number.

Our study has some limitations. For example, we did not exclude stroma from our analyses. The use of pattern-recognition software to identify and dial-out stromal data may therefore improve precision.

A recent study by Chabot-Richards *et al*. revealed that there is no substantial benefit to using quantitative image analysis for Ki-67 because of the good correlation between pathologist estimate and quantitative image analysis^33^. Their data suggested that pathologist visual scoring may be more closely associated with survival outcome than that of computerized image analysis. They explained that this may be caused by selecting appropriate areas of the slides by pathologists, however, the possible influence of unconscious recognition of nuclear atypia and cellularity by pathologists cannot be excluded. Since our new method reflects nuclear atypia and cellularity, and it has a good correlation with nuclear grade assessed by pathologist that associates prognosis, our new method may be more useful prognostic factor than the traditional image analysis for Ki-67.

In conclusion, this study demonstrates that PHI, a novel index for immunohistochemical labeling using persistent homology, can produce highly similar data to that generated by a pathologist using visual evaluation. The potential benefits associated with our novel technology include both improved quantification and reproducibility. Since our method reflects cellularity and nuclear atypia, it carries a greater quantity of biologic data compared to conventional evaluation using Ki-67.
